# Evaluation of Tear Evaporation Rate in Patients with Diabetes Using a Hand-Held Evaporimeter

**DOI:** 10.3390/healthcare10010104

**Published:** 2022-01-05

**Authors:** Ali Abusharha, Gamal A. El-Hiti, Mushawwat H. Alsubaie, Abdulaziz F. Munshi, Ahmed R. Alnasif, Raied Fagehi, Mana A. Alanazi, Ali M. Masmali

**Affiliations:** Department of Optometry, College of Applied Medical Sciences, King Saud University, P.O. Box 10219, Riyadh 11433, Saudi Arabia; gelhiti@ksu.edu.sa (G.A.E.-H.); mushawwata@gmail.com (M.H.A.); az.f.munshi@gmail.com (A.F.M.); ahmmed994@gmail.com (A.R.A.); rfagehi@ksu.edu.sa (R.F.); amana@ksu.edu.sa (M.A.A.); amasmali@ksu.edu.sa (A.M.M.)

**Keywords:** diabetes, dry eye symptoms, tear evaporation rate, lipids-rich artificial tears, evaporative dry eye

## Abstract

Diabetes is a very common disease and is considered a risk factor for many diseases such as dry eye. The aim of the current work was to evaluate the tear evaporation rate (TER) in patients with diabetes using a hand-held evaporimeter. This observational, case–control and non-randomized study included 30 male patients with diabetes (17 controlled and 13 uncontrolled) with a mean ± standard deviation (SD) of 33.1 ± 7.9 years. An age-matched (18–43 years; 32.2 ± 6.5 years) control group consisting of 30 male subjects was also enrolled for comparison. Subjects with thyroid gland disorder, a high body mass index, high blood cholesterol, or thalassemia, contact lens wearers, and smokers were excluded. The TER was measured after the completion of the ocular surface disease index (OSDI) by each participant. The OSDI and TER median scores were significantly (Wilcoxon test, *p* < 0.05) higher in patients with diabetes (median (interquartile range; IQR) = 12.0 (8.3) and 46.4 (36.7) g/m^2^h, respectively) compared to the subjects within the control group (5.6 (7.0) and 15.1 (11.9) g/m^2^h, respectively). The median scores for the OSDI and TER measurements were significantly (Wilcoxon test, *p* < 0.05) higher among uncontrolled diabetes patients (13.0 (11.5) and 53.4 (14.2) g/m^2^h, respectively) compared to those obtained for patients with controlled diabetes (11.0 (8.0) and 27.3 (32.6) g/m^2^h, respectively). The tear evaporation rate in patients with diabetes was significantly higher compared to those obtained in subjects without diabetes. Uncontrolled diabetes patients have a higher tear evaporation rate compared to controlled diabetes patients. Therefore, diabetes can lead to eye dryness, since these patients possibly suffer excessive tear evaporation.

## 1. Introduction

Diabetes results from high blood sugar due to a shortage or lack of insulin production [1]. Type 1 and type 2 are very common. Type 1 diabetes occurs due to a failure of insulin production owing to the destruction of pancreatic β-cells [2]. Conversely, type 2 diabetes develops from insufficient insulin secretion. Type 1 diabetes is more prevalent in children up to 14 years old [3], and type 2 diabetes is more prevalent in adults, especially those who are obese [4]. Diabetes is quite prevalent and can be acquired or inherited. Type 1 diabetes can decrease life expectancy by 12 years in comparison to the general population [5]. Currently, around 537 million adults are affected by diabetes [6]. The majority (79%) of patients with diabetes are living in middle- and low-income countries. It was estimated that around 10% of the world’s health expenditure (USD 760 billion) is spent on the treatment of diabetes. Additionally, the diabetes percentage is expected to increase in the next 20 years [6] and is projected to affect 629 million people by 2049 [7]. Currently, China, India, and the United States have the most cases of adult diabetes, that amounts to 224 million [6]. In 2019, the prevalence of diabetes was highest (12.2%) in the Middle East and North Africa (MENA) and lowest (4.7%) in Africa. It was estimated that 55 million individuals were diagnosed with diabetes in the MENA, with 4.3 million living in Saudi Arabia [6]. The number of deaths due to diabetes in the MENA is expected to be over 400,000 cases [8,9].

Lifestyle, diet, family history, vitamin D deficiency, and viral infections are the main contributors to the development of diabetes [10,11]. The consumption of sugary drinks and foods that are rich in carbohydrates and low in fibers could also contribute to the illness. Therefore, obesity is usually associated with a high prevalence of diabetes [12]. In addition, diabetes is associated with several other chronic illnesses [13,14,15,16,17] and many ocular complications (e.g., retinopathy, a reduced corneal sensitivity, a decrease in tear secretion, cataracts, corneal lesions, and glaucoma) [18,19,20,21,22]. In addition, altered healing of the corneal epithelium is a common corneal abnormality in patients with diabetes [23]. In animal models, the reduction in blood insulin levels can lead to a disturbance in culture maintenance, the proliferation of epithelial cells, and the lacrimal gland metabolism [24]. The disturbance in such ocular tissues might lead to eye dryness, mainly due to the dysfunction of lacrimal gland [24]. Additionally, alterations in the tear film could occur due to corneal nerve damage, chronic hyperglycemia, and insulin action impairment [25]. These ocular surface alterations that lead to dry eye symptoms in patients with diabetes have been previously studied [26,27,28,29].

Dry eye results from tear film instability due to a disturbance in tear secretion, tear evaporation, or both [30]. Eye dryness leads to various discomfort symptoms and causes ocular surface damage if not treated. Therefore, it is important to examine the ocular surface regularly to discover dry eyes early and avoid visual complications [31]. No single technique can be used to detect eye dryness, but a combination of different tests is necessary. The most common dry eye diagnostic tests are phenol red thread, Schirmer, tear meniscus height, tear break-up time, tear ferning, and osmolarity tests [32,33,34,35,36]. The tear evaporation rate (TER) test can be used to detect eye dryness and tear film instability, in combination with the other tests [37,38]. The test is simple, fast, repeatable, non-invasive, and convenient. Various techniques can be used to determine the TER rate with a normal eye up to 25 g/m^2^h (at a humidity of 30%). For dry eye, the TER is higher than 25 g/m^2^h [39,40]. Delfin VapoMeter (Delfin Technologies UK Limited, Surrey, UK) is a relatively new device that can measure the TER up to 200 g/m^2^h. It is portable, small, accurate, can be used at different angles with the eye either open or closed, and does not require daily calibration. Recently, Delfin VapoMeter was used to measure the TER in smokers and patients with thyroid gland disorder [37,38]. In the current research, the evaluation of the TER in patients with diabetes using Delfin VapoMeter is reported for the first time.

## 2. Materials and Methods

### 2.1. Subjects

This observational, case–control, and non-randomized study included 30 male patients with diabetes (17 controlled and 13 uncontrolled) with a mean ± standard deviation (SD) of 33.1 ± 7.9 years). An age-matched control group (18–43 years; 32.2 ± 6.5 years) consisting of 30 male subjects was also enrolled for comparison. The subjects were mostly students that have been recruited from King Saud University, Riyadh. Controlled and uncontrolled diabetes were diagnosed based on the hemoglobin A1c (HbA1c) test. An HbA1c level above 6.5% was defined as uncontrolled, while a level below 6.5% was considered controlled diabetes [41]. The participants were treated according to the tenets of the Declaration of Helsinki. Informed written consent was obtained from each subject before conducting the study. Subjects with thyroid gland disorder, a high body mass index (above 24.9 kg/m^2^), high blood cholesterol (above 4 mmol/L), or thalassemia (hemoglobin concentration is higher than 7 g/dL), contact lens wearers, and smokers were excluded. In addition, subjects with ocular and corneal surgeries, diseases, and disorders have been excluded from the study. The TER was measured after the completion of the ocular surface disease index (OSDI) by each participant. Measurement of the TER can be affected by the area of surface exposure, ambient airflow, blinking, humidity, and temperature [42]. The subjects were examined by the same researcher in an air-conditioned clinic in which the temperature (23 °C), the humidity (less than 40%), and airflow were controlled.

### 2.2. The OSDI

The OSDI score was calculated for each participant. A score greater than 12 was defined as dry eye disease [43].

### 2.3. The TER Test

A VapoMeter (Delfin Technologies UK Limited, Surrey, UK) was used to measure the TER. The measurements were carried out three times and averages were calculated. Two sets of measurements were performed, the first one with the participant’s eyes open and the second test with their eyes closed. A gap of two minutes was allowed between the two sets of tests. The TER rate was calculated by subtracting the scores obtained when both eyes were closed from the readings obtained when both eyes were open. A TER above 25 g/m^2^h was defined as dry eye disease, while a TER less than 25 g/m^2^h was defined as a normal eye.

### 2.4. Statistical Analysis

The data were collected and analyzed using Excel and SPSS^®^ statistical package (IBM, Armonk, NY, USA), version 22.0 for Windows^®^, respectively. The Kolmogorov–Smirnov test was used to test the normality of the data and the Wilcoxon test was used to the significance of the differences between scores.

## 3. Results

The subjects’ ages in the diabetes (33.1 ± 7.9 year) and the control (32.2 ± 6.5 year) groups were distributed normally (Kolmogorov–Smirnov test; *p* ˃ 0.05) and the average was presented as the mean ± SD. For the OSDI and TER scores, the data were abnormally distributed (Kolmogorov–Smirnov test; *p* < 0.05) and the average was presented as the median (interquartile range). The average ages and scores from the OSDI and TER measurements are shown in Table 1. The OSDI scores ranged from 3 to 34 and from 1 to 20 in the study and the control groups, respectively. The TER measurement ranged from 3.7 to 87.0 g/m^2^h and from 5.9 to 30.7 g/m^2^h in the study and the control groups, respectively. The TER measurements showed that the majority of study subjects (*n* = 22; 73.3%) have higher TER measurements (dry eye) compared to the readings recorded within the subjects in the control group. The OSDI score indicated eye dryness in half of the patients with diabetes (*n* = 15). There were significant (Wilcoxon test, *p* < 0.05) differences between the median scores obtained from the OSDI and the TER within the study and control groups. There was a medium correlation (*r* = 0.42; *p* = 0.03) between the scores from the OSDI and the TER measurements.

**Figure 1:**
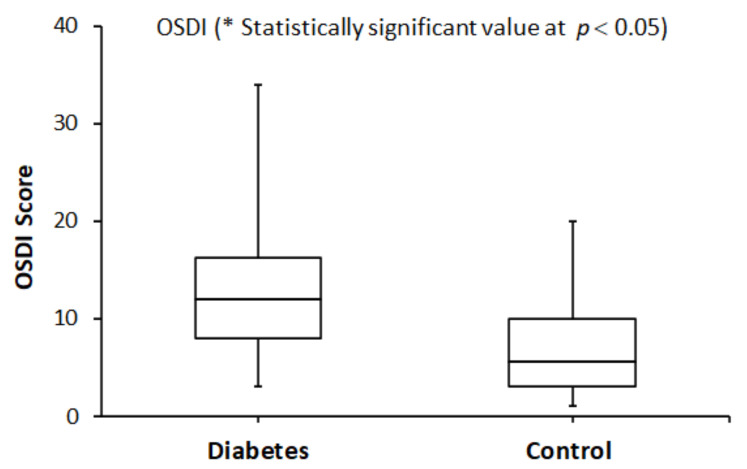
A side-by-side boxplot for the OSDI score within the study (diabetes) and the control (without diabetes) groups. * The difference in the OSDI median score was statistically significant at *p* < 0.05.

**Figure 2:**
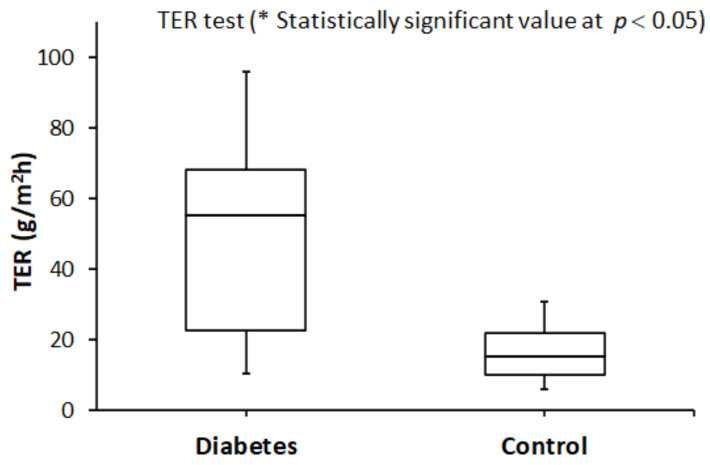
A side-by-side boxplot for the TER score within the study (diabetes) and the control (without diabetes) groups. * The difference in the TER median score was statistically significant at *p* < 0.05.

Side-by-side boxplots of the OSDI and TER scores in both groups are shown in Figure 1 and Figure 2, respectively. They clearly show that the median OSDI and TER scores were significantly higher (Wilcoxon test, *p* < 0.05) in the study group compared to that of the control group.

The study group (*n* = 30) included 17 controlled and 13 uncontrolled diabetes patients. The average ages, OSDI, and TER scores for the controlled and uncontrolled diabetes groups are shown in Table 2. For the OSDI, the median score was significantly higher (Wilcoxon test, *p* < 0.05) in the uncontrolled diabetes group compared to that of the controlled diabetes group. Similarly, the TER median score was significantly higher (Wilcoxon test, *p* < 0.05) in the uncontrolled diabetes patients compared to that of the controlled diabetes patients.

## 4. Discussion

The current study showed that most of the patients with diabetes (73.3%) have a significantly a higher TER than those without diabetes. Measurements of the TER in patients with diabetes have never been reported before and therefore no direct comparison can be made. The OSDI scores also demonstrated that 50% of the study group had eye discomfort. The prevalence of eye dryness and discomfort was higher in patients with uncontrolled diabetes compared to those with controlled diabetes. The current study suggests that diabetes has a negative effect on tear film stability, which is in agreement with previous studies. Therefore, diabetes is considered a risk factor for dry eye disease. An association between diabetes and dry eye disease has also been established previously [44,45]. In addition, diabetes leads to cataracts, glaucoma, diabetic retinopathy, refractory deviations, and an alteration in the healing of the corneal epithelium and the cornea’s sensitivity [23,46].

A higher prevalence of dry eye disease was found in women with diabetes (59%) than in men (49%) [45,46]. Additionally, the length of time that patients had diabetes was significantly associated (*p* = 0.01) with dry eye [46]. Examining the medical records of 22,382 patients with diabetes confirmed that 20.6% of them consumed significantly (*p* < 0.001) more ocular lubricants compared to the control group (13.8%) [27]. Keratoconjunctivitis sicca was found to be common in patients with diabetes [27]. Additionally, animals such as mice with diabetes developed dry eye symptoms as a result of lacrimal gland dysfunction [24].

A study conducted on 124 patients with controlled (*n* = 62) and uncontrolled (*n* = 62) diabetes (37.0 ± 7.0 years) showed a high prevalence for dry eye disease, especially among those with uncontrolled diabetes [47]. The scores collected from MacMonnies questionnaire showed that 23% of the subjects have dry eye discomfort symptoms in which the majority of them (82%) have uncontrolled diabetes [47]. The tear ferning grades obtained from the tears of patients with diabetes indicated that 35% of the subjects have dry eye disease, the majority of whom (72.7%) have uncontrolled diabetes [47]. The scores from the PRT and TBUT tests showed similar patterns [47]. Medium to strong negative (−0.349 to −0.539) correlations were found between the tear ferning grades and the PRT and TBUT test scores [47].

Another study on 50 patients with diabetes (54.6 ± 13.4 years) showed that half of the subjects have dry eye symptoms based on the OSDI and tear osmolarity test using the TearLab Osmolarity system [48]. Additionally, 50% of the patients with diabetes showed high tear osmolarity scores. The tear osmolarity measurements were higher in men (311.8 ± 4.0 mOsm/L) compared with women (302 ± 1.9 mOsm/L) [48]. A study on 88 patients with diabetes (38 men and 50 women) aged 31 to 77 years (55.0 ± 10.1 year) showed higher dry symptoms in the patients with diabetes compared to those without diabetes (*n* = 88) [49]. The median scores from the TBUT and tear meniscus height tests were lower in the patients with diabetes (5.0 (2.0) s and 0.6 (0.6), respectively)) compared to those obtained in subjects without diabetes (7.0 (3.0) s and 0.8 (0.7), respectively). The corneal staining average score was higher in the patients with diabetes (0.5 (2.0) mm) than in those without diabetes (0.0 (1.0) mm) [49].

In another study, the corneal sensitivity, TBUT, and Schirmer tests average scores in non-insulin-dependent patients with diabetes (*n* = 50; 54.2 year) were significantly lower (*p* < 0.001) compared to the average scores obtained from the control group (*n* = 20; 56.4 year) [28]. The mean scores for the corneal sensitivity, TBUT, and Schirmer tests within the study group were 44.0 ± 1.1 mm, 8.8 ± 0.3 s, and 7.4 ± 0.4 mm, respectively, and in the control group, they were 59.4 ± 0.6 mm, 13.0 ± 1.4 s, and 13.5 ± 0.5 mm, respectively [28]. A study conducted on insulin-dependent patients with diabetes (*n* = 86; 58.0 ± 8.0 year) showed that tear flow and tear volume, based on the Schirmer tests, were lower (1.1 ± 0.4 μL/min and 10 ± 3 mm, respectively) compared to those obtained from the control group (60.0 ± 9.0 year; 1.1 ± 0.4 μL/min, and 10.0 ± 3.0 mm, respectively) [29]. The mean TBUT test score was higher in patients with diabetes (18.0 ± 10.0 s) compared with the control group (16.0 ± 11.0 s) [29]. The difference between the tear volume within the study and control group was significant (*p* < 0.001). However, no significant differences were found between the groups with respect to the tear flow and TBUT [29]. In addition, a recent study showed no significant (*p* = 0.747) difference in the TBUT measurements in subjects with debates and those in the control group [50]. On the other hand, a more recent study indicated that Indian subjects with diabetes have significantly (*p* < 0.001) lower TBUT measurements (9.7 ± 2.9 s) compared with those in the control group (14.5 ± 2.9 s) [51]. Such a finding is in contrast with studies conducted earlier.

Recently, the TER in chronic smokers and patients with thyroid gland disorder were measured using a VapoMeter [37,38]. The TER median score of 20 patients with thyroid gland disorder (34.3 ± 6.3 year) was significantly (*p* < 0.05) higher (41.2 (41.4) g/m^2^h) than that of the control group (*n* = 20; 32.2 ± 5.1 year; 15.7 (13.7) g/m^2^h) [37]. A similar observation was seen for the TER among smokers [38]. The TER median score for 120 smokers studied (24.4 ± 5.8 year) was significantly (*p* < 0.05) higher (37.7 (59.3) g/m^2^h) than that of the control group (*n* = 120; 26.1 ± 5.2 year; 15.4 (13.1) g/m^2^h) [38].

In patients with diabetes, the reduction in insulin levels can disturb the biomechanical balance of body tissues, possibly leading to dry eye symptoms [39]. The high TER (>25 g/m^2^h) in patients with diabetes might be due to a reduction in the blinking rate [39]. Moreover, the disruption in the structure of the tear film lipid layer plays an important role and tends to increase the TER by ~90–95% in chronic smokers and patients with thyroid gland disorder. The lipid layer thickness is inversely related to the TER [37,38]. Patients with diabetes have a lower lipid layer thickness [52]. The disturbance in the lipid layer thickness of the tear film leads to a change in the blinking rate [53]. Blinking is responsible for the spread and distribution of lipid across the tear film. The disturbance in blinking rate causes tear film instability and induced symptoms of dry eye, mainly due to the excessive evaporation of tears [54].

The limitations of the study include the recruitment of a relatively small number of young subjects, no females have taken part, and only one location (Riyadh City) was covered.

## 5. Conclusions

The tear evaporation rate in patients with diabetes was significantly higher compared to those obtained in subjects without diabetes. Uncontrolled diabetes patients have a higher tear evaporation rate compared to controlled diabetes patients. Therefore, diabetes can lead to eye dryness, since it possibly leads to excessive tear evaporation.

## Figures and Tables

**Table 1:** The averages (mean ± SD or median (IQR)) for the age, OSDI, and TER scores within the study and the control groups.

Score	Diabetes Group (*n* = 30)	Control Group (*n* = 30)
Age (year)	33.1 ± 7.9	32.2 ± 6.5
OSDI *	12.0 (8.3)	5.6 (7.0)
TER (g/m^2^h) *	46.4 (36.7)	15.1 (11.9)

* Statistically significant value at *p* < 0.05. SD: standard deviation, IQR: interquartile range.

**Table 2:** The averages (mean ± SD or median (IQR)) for the age, OSDI, and TER scores within controlled and uncontrolled diabetes.

Score	Controlled Diabetes (*n* = 17)	Uncontrolled Diabetes (*n* = 13)
Age (year)	28.8 ± 7.8	37.3 ± 5.3
OSDI *	11.0 (8.0)	13.0 (11.5)
TER (g/m^2^h) *	27.3 (32.6)	53.4 (14.2)

* Statistically significant value at *p* < 0.05; the level of HbA1c is below 6.5% for controlled diabetes and above 6.5% for uncontrolled diabetes. SD: standard deviation, IQR: interquartile range.

## Data Availability

Data are contained within the article.
